# 5^th^ International AIDS Society Conference on HIV Pathogenesis, Treatment and Prevention: summary of key research and implications for policy and practice – Biomedical prevention

**DOI:** 10.1186/1758-2652-13-S1-S4

**Published:** 2010-06-01

**Authors:** Mark Mascolinli, Rodney Kort

**Affiliations:** 1 Allentown, 18102, USA; 2 Kort Consulting, Toronto, M4Y 2T6, Canada

## Abstract

No major findings were reported at the 5^th^ IAS Conference on HIV Pathogenesis, Treatment and Prevention (IAS 2009) on currently enrolled microbicides, pre-exposure prophylaxis (PrEP) or vaccine trials, although important findings in all three areas of biomedical prevention research are expected within the next few years.

A study found that daily acyclovir did not reduce HIV transmission, but was a factor in modest reductions in viral load, which could confer some clinical benefit. Research demonstrating rapid viral replication in mucosal tissue and subsequent dissemination throughout the body suggested that research priorities should shift towards a mucosal vaccine. Findings reported in Track C indicated that, in addition to reducing vertical transmission, antiretroviral therapy (ART) also lowers the risk of prematurity, stillbirth and abortion.

Challenging concerns about the potential “disinhibiting” effect of ART as prevention, a Kenyan study found that widespread ART encourages greater use of condoms and does not increase the rate of risky sex. Another Kenyan study found that pregnancy increases the risk of HIV transmission in a cohort of serodiscordant couples. Although three randomized trials have conclusively demonstrated that circumcision reduces HIV transmission among heterosexual men, research presented at IAS 2009 found no evidence of a preventive impact for women.

## Discussion

IAS 2009 included no major reports on three biomedical prevention fronts (microbicides, PrEP and vaccines), although important Phase III microbicide and PrEP trials are under way and evidence is mounting regarding the impact of ART rollout on reducing HIV transmission (see Additional File 1). On the vaccine front, Track C lead rapporteur Sinead Delany-Moretlwe [1] (University of Witwatersrand, Johannesburg) reviewed an analysis by José Esparza (Bill & Melinda Gates Foundation, Seattle), who noted that systemic immunity to HIV-1 rarely occurs in nature and may not be possible with a vaccine [2]. Recent work demonstrating rapid viral replication in the mucosa and rapid systemic dissemination suggests research priorities should shift to a mucosal vaccine that elicits an immune response at the viral portal of entry, Esparza argued. The meeting did feature important studies on prevention with ART, circumcision and acyclovir.

### ART impact on pregnancy outcome and risky sex

Triple-drug ART does more than prevent vertical transmission (as outlined in the Track B discussion); an observational study of 3273 pregnant women in Malawi and Mozambique showed that it also lowers the risk of prematurity, abortion and stillbirth among African women, providing additional insights into the benefits of ART [3]. This study recorded significantly lower mortality among pregnant women who started nevirapine-based ART than in those who did not (0.7% versus 7.4%, P <0.001).

Leonardo Palombi (DREAM Program, Rome) reported that rates of abortion or stillbirth were 4.3% in the ART group and 25.7% in the no-ART group (P <0.001). Regardless of CD4 cell stratum, the prematurity rate was 70.8% lower in women who took antiretrovirals.

A prospective study of 898 female bar workers in Mombasa, Kenya, found no evidence supporting the hypothesis that widespread ART will encourage more risky sex in low-income countries. Indeed, women in this cohort reported safer behaviour after they began ART [4] than before they started. Linnet Masese (University of Nairobi) found significantly higher rates of 100% condom use after ART began and a strong trend toward abstinence or having only one sex partner during ART. Sexually transmitted infections were one-third less likely after ART began.

### Pregnancy doubles serodiscordant HIV transmission risk

**Figure 1:**
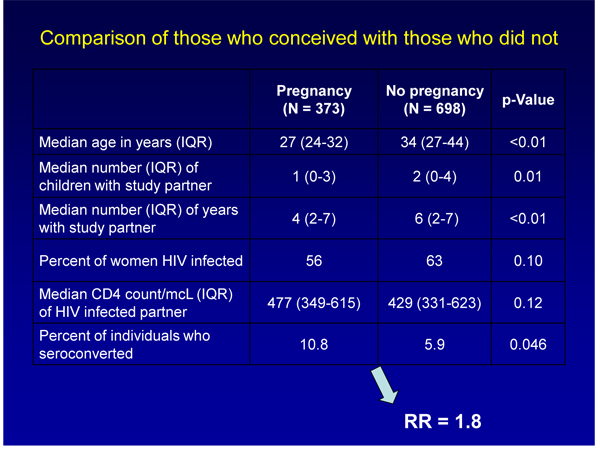
**Pregnancy and the risk of HIV transmission**. Source: Brubaker S, et al. **Pregnancy and HIV transmission among HIV discordant couples in a clinical trial in Kisumu, Kenya.** 5th IAS Conference on HIV Pathogenesis, Treatment and Prevention: Cape Town, South Africa. WELBC105 [5].

Pregnancy doubled the risk of HIV transmission in a two-year study of more than 500 HIV-discordant couples (one positive partner, one negative partner) in Kisumu, Kenya [5]. Sara Brubaker (University of California, San Francisco) found that among 539 female partners (95% of them married), 189 (35%) conceived. Forty-one of 539 uninfected partners (8%) became infected during the study (see Figure 1). Among women who conceived or men whose partners conceived, 10.8% became infected during follow up, compared with 5.9% of partners who did not have a child.

Those rates translated into a 1.8 times higher risk of HIV infection in couples who had a child (P=0.046). About 30% of new HIV infections happened six months before conception, another 35% six months after conception, and the remaining 35% more than six months from conception. The presenting author noted that the data did not determine intentionality to conceive and that – if this is indeed the case – couples may be risking a greater probability of HIV transmission in order to conceive.

### Circumcision and HIV risk in female partners

**Figure 2:**
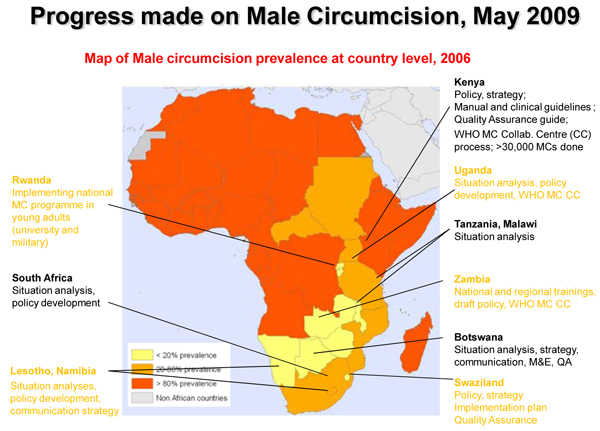
**Progress on male circumcision.** Source: Sinead Delany-Moretlwe: **Track C Rapporteur Report.** 5th IAS Conference on Pathogenesis, Treatment and Prevention: Cape Town, South Africa. [1]

Three randomized trials conclusively demonstrate that circumcision lowers risk of HIV acquisition in heterosexual men, and circumcision programmes are being rolled out in a number of countries in sub-Saharan Africa (see Figure 2) [6-8]. But how the procedure affects HIV risk in female partners remains unknown. Results from two studies of serodiscordant couples (an observational study presented at IAS 2009 and a randomized controlled trial published in the scientific literature) could not establish that circumcision protects female partners from HIV.

The prospective observational study weighed the risk of HIV transmission in 1096 serodiscordant couples with HIV-positive male partners who were enrolled in a trial of acyclovir to prevent HIV transmission [9]. During one to two years of follow up at 14 sites across Africa, 64 female partners became infected; viral sequencing established the male partners as the source in 50 of these women. One-third of the men were circumcised. Jared Baeten (University of Washington, Seattle) found that the overall new infection rate was 2.7 per 100 person-years in female partners of circumcised men versus 4.4 in partners of uncircumcised men. Among newly infected female partners whose HIV sequences matched that of their partners, HIV incidence was 2.0 in partners of circumcised men and 3.5 in partners of uncircumcised men. In both of these comparisons, the risk of HIV infection adjusted for male viral load fell short of statistical significance (P=0.13).

A trial that randomized HIV-infected Ugandan men to immediate or deferred circumcision found no significant difference in HIV incidence among partners of circumcised men (18%) and uncircumcised men (12%) [10]. The investigators note that their analysis cannot exclude “the possibility of higher risk of transmission in couples who resumed sexual intercourse before complete [circumcision] wound healing”.

### Daily acyclovir and risk of HIV transmission or progression

Two previously published placebo-controlled trials of daily acyclovir found that this antiherpes drug does not prevent HIV acquisition in women and men infected with herpes simplex virus type 2 (HSV-2) [11,12]. A trial presented at IAS 2009 found that daily acyclovir does not prevent HIV transmission from HIV/HSV-2 infected people to their partners [13]. This trial, involving 3408 heterosexual HIV-discordant African couples at 14 sites, randomized HIV-1/HSV-2 coinfected partners to placebo or to 400mg of acyclovir twice daily. Two-thirds of coinfected partners were female, no one was taking antiretrovirals, and participants reported taking 96.5% of acyclovir doses.

Connie Celum (University of Washington, Seattle) reported that after 24 months of follow up, 84 new HIV-1 infections could be linked to study partners, 41 of them taking acyclovir and 43 taking placebo. Daily acyclovir significantly lowered the frequency of HSV-2-positive genital ulcers and reduced HIV load by an average 0.25 log, but those benefits were insufficient to protect partners from HIV transmission.

Although the modest decline in viral load recorded in this trial did not prevent coinfected partners from transmitting HIV to their partners, even a small decline may confer clinical benefits, according to secondary results of another African study [14]. This placebo-controlled trial involved 2284 women and 1097 men coinfected with HIV-1 and HSV-2, but taking no antiretrovirals because they had a CD4 count above 250 cells/mm^3^. Jairam R Lingappa (University of Washington, Seattle) found that 284 people taking acyclovir and 325 taking placebo reached a composite endpoint of a CD4 count below 200 cells/mm^3^, ART initiation, or death from causes other than trauma. Those numbers translated into a 27% lower risk of reaching the composite endpoint (P=0.03).

## Conclusions

The 3000-woman analysis demonstrating lower rates of prematurity, abortion and stillbirth in women taking a triple-antiretroviral combination [3] adds to the already ample evidence supporting standard ART during pregnancy and breastfeeding. At this point, however, many pregnant or breastfeeding women do not receive standard ART because they do not meet national treatment criteria. In female Mombasa barmaids who reported transactional sex, starting ART coincided with less high-risk sexual behaviour [4], a finding suggesting that ART does not have a “sexual disinhibiting” effect, at least in this population. These results also confirm the feasibility of bringing high-risk women into care.

The study finding a higher risk of HIV transmission in HIV-discordant couples who have a child [5] indicates that discordant partners may continue to conceive even when they know one partner has HIV. If a portion of these pregnancies were intentional, the investigators suggest, these couples are risking HIV transmission in order to conceive. The results emphasize the importance of targeting serodiscordant married and unmarried couples in prevention initiatives.

Two studies failed to establish a lower risk of HIV infection in female partners of circumcised versus uncircumcised men [9,10]. The authors of the study presented at IAS 2009 suggest “short term interventions to protect against transmission risk during wound healing after circumcision of HIV-1 infected men could be considered, in order to realize the longer term potential benefits of male circumcision” [9]. The authors of a previously published randomized trial add that protecting men from becoming infected by circumcising them probably lowers overall HIV transmission risk in a heterosexual population because it lowers the proportion of men carrying HIV [10]. There is no doubt that circumcision can be a valuable facet of HIV prevention for heterosexual men. (There have been no randomized trials of circumcision in men who have sex with men.)

The acyclovir trials show that this antiherpes agent has no role in preventing HIV transmission [13], but it can help slow HIV disease progression in people not taking ART [14]. The latter finding emphasizes the importance of testing simple, inexpensive non-ART strategies, such as acyclovir and cotrimoxazole, in people with HIV.

Track C lead rapporteur Sinead Delany-Moretlwe drew four lessons from prevention studies presented at the conference [1]:

• Treatment has progressed; prevention remains challenging.

• Biomedical interventions still require behavioural change; risk compensation needs to be monitored.

• The concept of treatment for prevention brings the treatment and prevention worlds closer than ever before.

• New interventions for prevention are most likely to be effective when delivered in combination.

## Competing interests

Mark Mascolini and Rodney Kort are independent consultants contracted by the International AIDS Society for the purpose of drafting the IAS 2009 Impact Report: Summary of Key Research and Implications for Policy and Practice.

## Authors’ contributions

MM drafted the initial text and RA provided editorial input and advice. RK adapted the text for publication in a peer-reviewed journal. Both authors have approved the manuscript for publication.

## Supplementary Material

Additional file 1. New data on the preventive impact of antiretroviral therapy Click here for file
