# Supplementary material for: 5th International AIDS Society Conference on HIV Pathogenesis, Treatment and Prevention: summary of key research and implications for policy and practice – Biomedical prevention
Source: J Int AIDS Soc. 2010 Jun 1;13(Suppl 1):S4. doi: 10.1186/1758-2652-13-S1-S4 (PMC2880255; doi:10.1186/1758-2652-13-S1-S4)
Supplement: Additional file 1. New data on the preventive impact of antiretroviral therapy [file 1758-2652-13-S1-S4-S1.doc]

# Additional file: New data on the preventive impact of antiretroviral therapy

IAS 2009 offered an overview of and several new insights into the potential role of antiretroviral therapy to prevent HIV transmission. In a plenary address, Reuben Granich (World Health Organization, Geneva) argued that the rationale for ART as a prevention strategy is irrefutable:[1]

- Transmission only occurs from persons with HIV.
- Viral load is the single greatest risk factor for HIV transmission.
- ART can lower viral load to undetectable levels.
- Prevention of vertical transmission is proof of concept that ART reduces transmission.
- Observational evidence in heterosexual couples supports the concept.
- Previous modelling work suggests considerable potential.

Granich’s own modelling study determined that treating all HIV-infected people with a CD4 count below 350 cells/mm3 would save 2.4 million lives between now and 2050, while universal voluntary testing and immediate ART would save 7.35 million lives.[2]

Other research on the preventive impact of ART yielded the following findings:

A study of 2993 HIV-discordant couples in Rwanda and Zambia recorded four HIV infections from partners on ART and 171 from partners not on ART (incidence 0.7% versus 3.4%).[3]

A modelling study of male circumcision, condom use and ART in South Africa determined that 50% ART coverage would prevent 371,370 infections over 20 years, 75% coverage would prevent 635,790, and 90% coverage would prevent 770,330.[4] These prevention rates were greater than those attained with circumcision and less than those attained with condom use.

**References**

1. **Granich R: HAART as prevention. *5th IAS Conference on HIV Pathogenesis, Treatment and Prevention*: 19 – 23 July 2009, Cape Town, South Africa. MOPL101.**
2. [Granich RM](http://www.ncbi.nlm.nih.gov/pubmed/1903848), Gilks CF, Dye C, De Cock KM, Williams BG: [**Universal voluntary HIV testing with immediate antiretroviral therapy as a strategy for elimination of HIV transmission: a mathematical model.**](http://www.ncbi.nlm.nih.gov/pubmed/19038438?ordinalpos=5&itool=EntrezSystem2.PEntrez.Pubmed.Pubmed_ResultsPanel.Pubmed_DefaultReportPanel.Pubmed_RVDocSum) *Lancet*. 2009; 373:48-57.
3. Sullivan P, Kayitenkore K**,** Chomba E, Karita E, Mwananyanda L, Vwalika C, Conkling M, Luisi N, Tichacek A, Allen S: **Is the reduction of HIV transmission risk while prescribed antiretroviral therapy (ARVT) different for men and women? Results from discordant couples in Rwanda and Zambia. *5^th^ IAS Conference on HIV Pathogenesis, Treatment and Prevention*: Cape Town, South Africa. WEAC101.**
4. Lima V, Anema A, Wood R, Moore D, Harrigan R, Mills E, Hogg R, Montaner J: **The combined impact of male circumcision, condom use and HAART coverage on the HIV-1 epidemic in South Africa: a mathematical model. *5^th^ IAS Conference on HIV Pathogenesis, Treatment and Prevention*: Cape Town, South Africa.** [WEAC105](http://www.ias2009.org/PAGMaterial/WEAC105_Lima_1.ppt)**.**
